# Supplementary material for: Pressure-stabilized divalent ozonide CaO3 and its impact on Earth’s oxygen cycles
Source: Nat Commun. 2020 Sep 17;11:4702. doi: 10.1038/s41467-020-18541-2 (PMC7499259; doi:10.1038/s41467-020-18541-2)
Supplement: Supplementary file 1 — Supplementary Information [file 41467_2020_18541_MOESM1_ESM.pdf]

Supplementary Information

Pressure-stabilized divalent ozonide CaO_3 and its impact on Earth's oxygen cycles

Wang et al.

Supplementary Note

Our structure prediction is performed using CALYPSO (Crystal structure AnaLYsis by Particle Swarm Optimization) methodology[1] as implemented in its same-name CALYPSO code[2] (CALYPSO code is free for academic use, by registering at http://www.calypso.cn.), which is based on a global minimization of free energy surfaces in conjunction with *ab initio* total-energy calculations. The CALYPSO approach has been used to predict structures of a broad range of materials including isolated clusters or nanoparticles, two-dimensional layers or reconstructed surfaces, and three-dimensional bulks, at ambient or high pressure conditions, with a variety of functional properties[3,4]. The effectiveness of the CALYPSO method has been demonstrated by the successful applications in predicting structures of many systems, ranging from elemental to binary and ternary compounds[1]. Prominent examples include high-pressure structures of lithium[5], bismuth telluride[6], water ice[7], cage-like diamondoid nitrogen[8], Xe-Fe[9], H_2S[10] and rare-earth hydrides[11], among which the high-pressure insulating *Aba*2-40 (Pearson symbol oC40) structure of lithium, two low-pressure monoclinic structures of bismuth telluride and Cu_3Fe-type $XeFe_3$ structure have been confirmed by experiments[12–14].

In this work, structure searches were performed at 30 and 50 GPa for Ca_mO_n (m=1, 2 and n=2, 3, 4) with one to four formula units. Each generation contained 30 structures, and the first generation was produced randomly with symmetry constraints. All structures were locally optimized using the VASP code[15]. Local optimizations performed during structure search were done with the conjugate gradients method and stopped when enthalpy changes became smaller than 1×10^{-5} eV per cell. The 60% lowest-enthalpy structures of each generation were used to produce the structures in the next generation by local PSO techniques, and the remaining 40% structures were randomly generated within symmetry constraints to enhance structural diversity. The search process was terminated after generating 600 structures at each run.

Structural optimization, electronic structure and phonon calculations were performed in the framework of density functional theory within the generalized gradient approximation[16] as implemented in the VASP code[15]. The electron-ion interaction was described by the projector augmented-wave potentials[17], with $3s^2 3p^6 4s^2$ and $2s^2 2p^4$ configurations treated as the valence electrons of Ca and O, respectively. A kinetic cutoff energy of 1,000 eV and a spacing of $2\pi \times 0.03$ Å^{-1} for Monkhorst-Pack k-mesh sampling[18] were adopted to give well converged total energies (~1 meV/atom). The ionic positions were fully relaxed until the residual force acting on each ion was less than 0.01 eV/Å. The dynamic stability of the predicted new phases was verified by phonon calculations using the direct supercell method as implemented in the PHONOPY code[19]. The Heyd−Scuseria−Ernzerhof (HSE) hybrid functional[20] was employed to get accurate electronic properties. The electronic density of states has been calculated using a large Monkhorst-Pack k point set generated from a 7 × 7 × 11 k mesh for CaO_3 and hypothetical Ca_0O_3. To determine charge transfer, we used the Bader charge analysis[21]. For the calculations of vibrational normal modes, the simulations of the finite displacements were run using the VASP code and Raman activity of modes were analyzed by vasp_raman.py[22,23].

We examined the following reactions leading to the formation of CaO_3:

$$CaO+O_2=CaO_3, \quad (1)$$

$$Ca(OH)_2+O_2=H_2O+CaO_3, \quad (2)$$

$$Ca(OH)_2+Al_2O_3+O_2=2AlOOH+CaO_3, \quad (3)$$

$$4FeO_2+CaO+2H_2O=4FeOOH+CaO_3. \quad (4)$$

The formation enthalpy of CaO_3 in Supplementary Equation 1 was determined by $\Delta H = [H(CaO_3) - H(CaO) - H(O_2)]$, where H is the enthalpy of the most stable structure of each composition at a given pressure. For CaO and O_2, face-centered cubic[24] and *Cmcm* structure[25] were used, respectively. The reaction enthalpy in Supplementary Equation 2 was obtained by $\Delta H = [H(Ca(OH)_2) + H(O_2) - H(H_2O) - H(CaO_3)]$. The $Ca(OH)_2$-II[26],

Cmcm[25] and VIII-ice[27] structures were used for $Ca(OH)_2$, O_2 and H_2O, respectively. The reaction enthalpy of Supplementary Equation 3 was obtained by $\Delta H = [H(Ca(OH)_2) + H(Al_2O_3) + H(O_2) - H(2AlOOH) - H(CaO_3)]$. The $Ca(OH)_2$-II[26], corundum phase[28], *Cmcm*[25] and δ-AlOOH[29] structures were used for $Ca(OH)_2$, Al_2O_3, O_2 and AlOOH, respectively. The reaction enthalpy of Supplementary Equation 4 was obtained by $\Delta H = [H(FeO_2) + H(CaO) + H(2H_2O) - H(2FeOOH) - H(CaO_3)]$. The pyrite-type[30], simple cubic[24], X-ice[31], and pyrite-type[32] structures were used for FeO_2, CaO, H_2O, and FeOOH, respectively. Structural details are listed in Supplementary Table 1.

Supplementary Table 1. Structural parameters of various structures studied in this work.

Pressure (GPa)	System	Space group	Lattice Parameters (Å,°)	Atom	Atomic coordinates (fractional) X	Y	Z
35	CaO_3	*P*-42$_1$*m*	a=4.87<br>c=2.98	Ca(2a)	0.00000	0.00000	0.00000
				O(4e)	0.82587	0.67413	0.52322
				O(2c)	0.50000	0.00000	0.21836
35	CaO_4	*I*4/*mcm*	a=5.05<br>c=7.17	Ca(4a)	0.00000	0.00000	0.25000
				O(16l)	0.14431	0.35569	0.09081
50	CaO_4	*P*2$_1$/*c*	a=3.57<br>b=5.69<br>c=4.18<br>β=104.83	Ca(2b)	0.50000	0.00000	0.00000
				O(4e)	0.08230	0.22159	0.63009
				O(4e)	0.73029	0.36302	0.06052
30	KO_3	*I*4/*mcm*	a=7.89<br>c=5.74	K(4a)	0.00000	0.00000	0.00000
				K(4b)	0.50000	0.00000	0.75000
				O(8h)	0.27169	0.22831	0.00000
				O(16k)	0.24315	0.06073	0.00000
40	$Ca(OH)_2$	*P*2$_1$/*c*	a=4.48<br>b=5.77<br>c=5.35<br>β=97.66	Ca(4e)	0.32598	0.06334	0.19387
				O (4e)	0.13279	0.40396	0.26941
				O (4e)	0.34535	0.78703	0.50128
				H (4e)	0.19108	0.52990	0.15702
				H (4e)	0.14154	0.78511	0.40216
20	Al_2O_3	*R*-3*c*	a=4.70<br>c=12.78<br>γ=120.00	O(18e)	0.30684	0.00000	0.25000
				Al(12c)	0.00000	0.00000	0.35201

			a=4.60	O (4g)	0.35150	0.23922	0.00000
20	AlOOH	*Pnnm*	b=4.09	Al (2a)	0.00000	0.00000	0.00000
			c=2.78	H (2d)	0.50000	0.00000	0.00000
			a=4.48				
80	FeO_2	*Pbca*	b=5.77	Fe(4b)	0.00000	0.00000	0.50000
			c=5.35	O (8c)	0.64636	0.64831	0.14573
20	CaO	*Fm*-3*m*	a=4.61	Ca (4a)	0.00000	0.00000	0.00000
				O (4b)	0.50000	0.50000	0.50000
80	CaO	*Pm*-3*m*	a=2.62	Ca (1a)	0.00000	0.00000	0.00000
				O (1b)	0.50000	0.50000	0.50000
			a=4.62	Fe(4b)	0.00000	0.50000	0.00000
80	FeOOH	*Pbca*	b=4.59	O (8c)	-0.35682	0.35772	0.14675
			c=4.61	H (4a)	0.00000	0.50000	0.50000
30	H_2O	$I4_1/amd$	a=4.14	O(8e)	0.50000	0.50000	0.24042
			c=5.92	H(16h)	0.50000	0.69584	0.34272
80	H_2O	*Pn*-3*m*	a=2.70	O(2a)	0.0000	0.00000	0.0000
				H(4c)	0.75000	0.75000	0.7500
			a=6.45				
30	O_2	*Cmcm*	b=3.47	O(8g)	0.09325	0.29003	0.25000
			c=2.84				
			a=7.70	O(8j)	0.53167	0.23490	0.68624
50	O_2	*C*2/*m*	b=4.51	O(4i)	0.19899	0.00000	0.31453
			c=3.47	O(4i)	0.26232	0.00000	0.68730

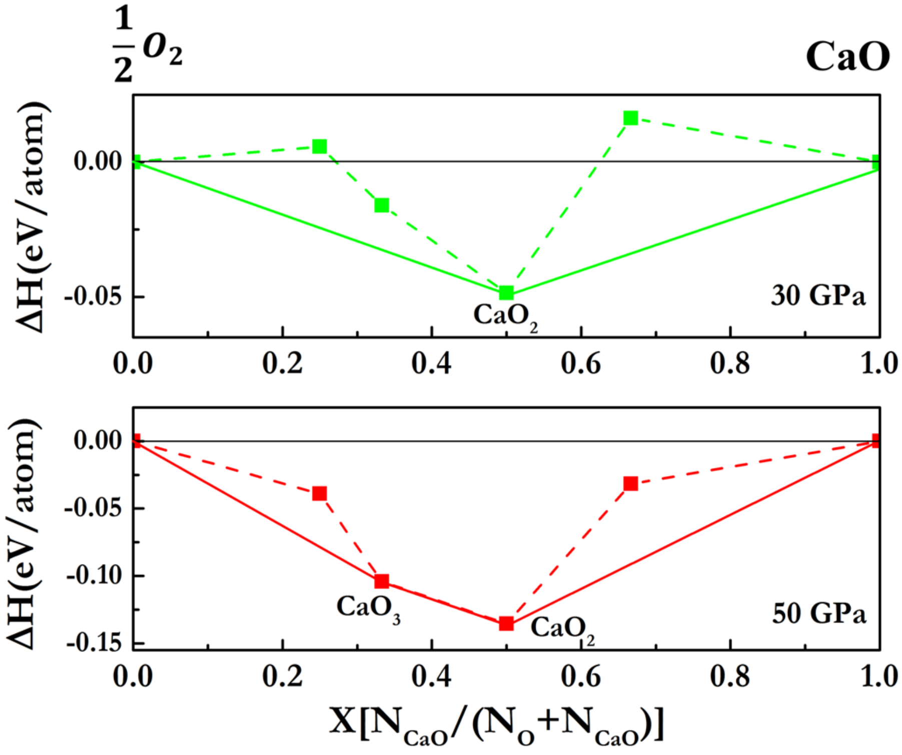

Supplementary Figure 1| The convex hull of the CaO-O_2 systems. Predicted formation enthalpy of various Ca–O compounds with respect to decomposition into CaO in *Fm*-3*m* structure and solid O_2 in *Cmcm* and *C*2*m* structures at 30 GPa and 50 GPa, respectively. Dashed lines connect data points, and solid lines denote the convex hull.

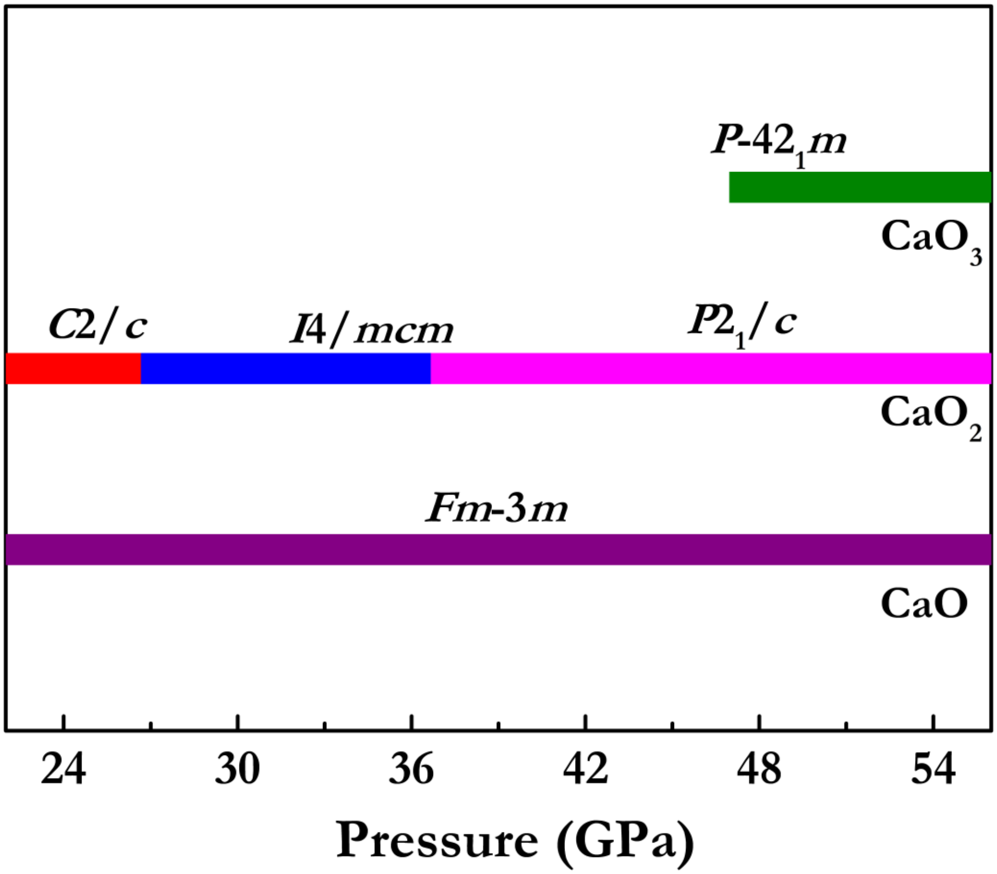

Supplementary Figure 2| Pressure-composition phase diagram of the Ca-O system. The tetragonal CaO_3 phase is predicted to be stable with respect to CaO_2 and solid O_2 above 47.8 GPa. For CaO_2, the *C*2*c* phase is predicted to be stable up to 27.2 GPa, followed by the tetragonal phase that goes through a phase transition to monoclinic structure ($P2_1/c$) at 37.2 GPa. These predicted high-pressure structures and their phase transition pressures are in good agreement with previous theoretical calculations[33], validating the reliability of our calculations.

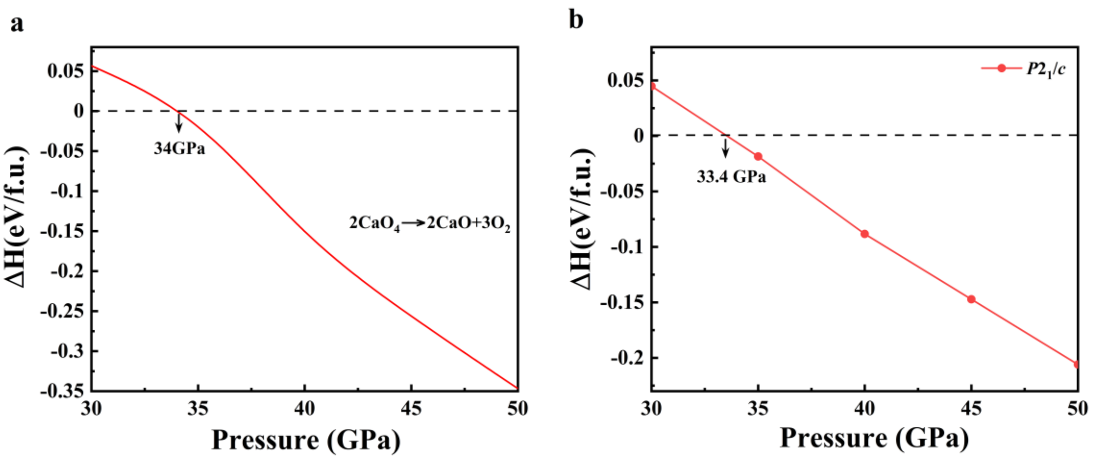

Supplementary Figure 3| Energetics of CaO_4 at high pressure. (**a**) The formation enthalpies of CaO_4 with respect to decomposition into CaO and solid O_2 and (b) The enthalpies of predicted crystal structure of $P2_1/c$ relative to the $I4/mcm$ phase of CaO_4.

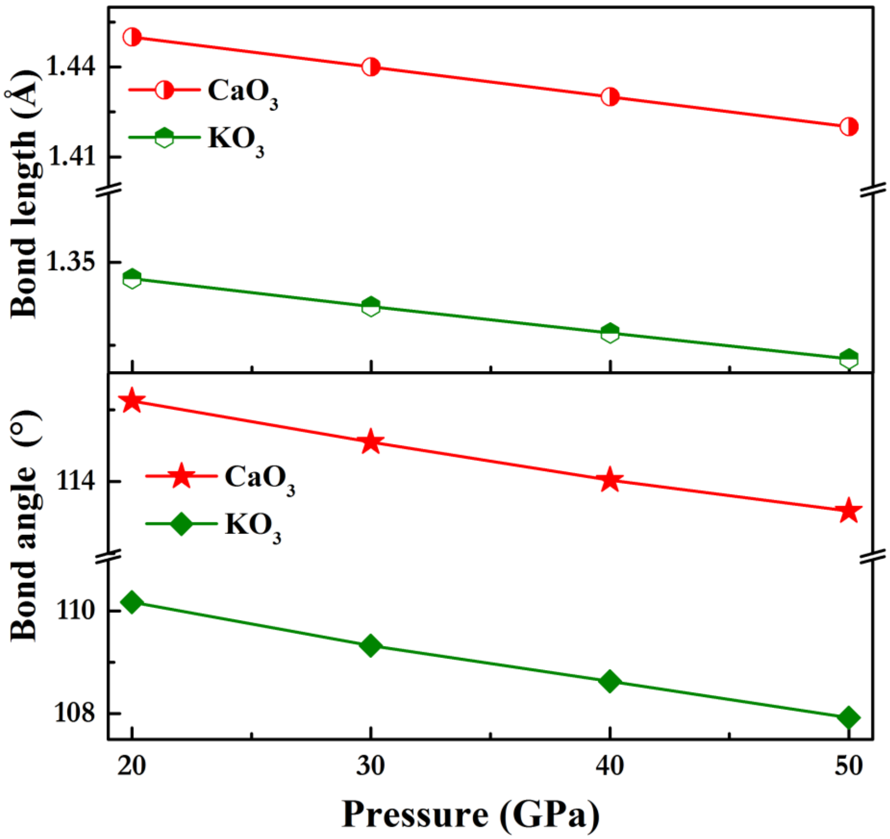

Supplementary Figure 4| Pressure dependence of the bond lengths and angles for CaO_3 and KO_3. The O-O bond length and O-O-O bond angle of the ozonide anion in CaO_3 are significantly larger than that in KO_3 at pressures ranging from 20 to 50 GPa.

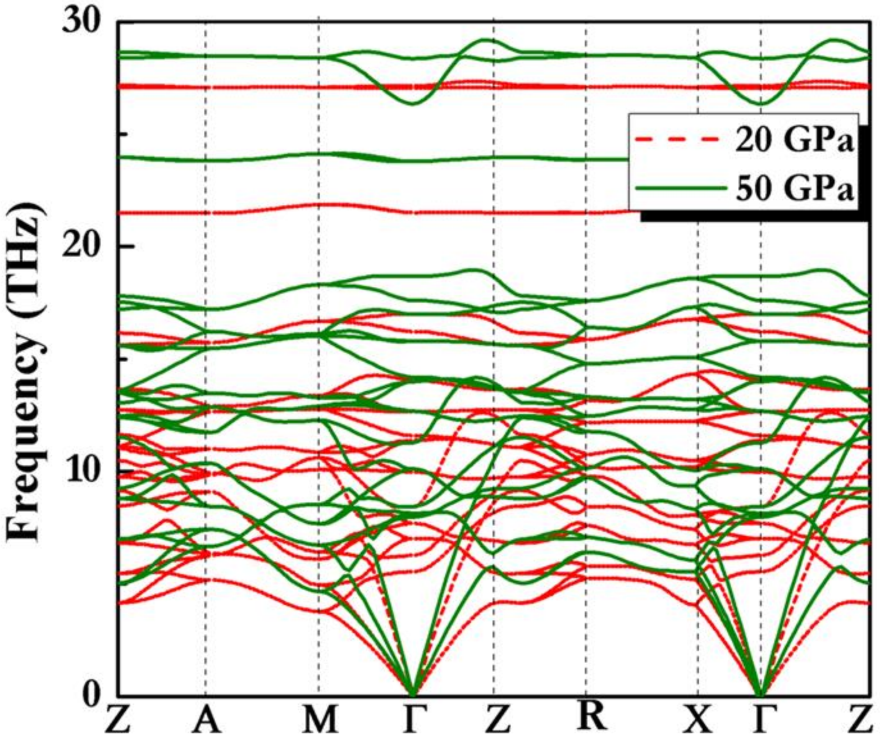

Supplementary Figure 5| Dynamic stability of CaO_3. Phonon dispersion relations along select high-symmetry points in the Brillouin zone for CaO_3 at 20 and 50 GPa.

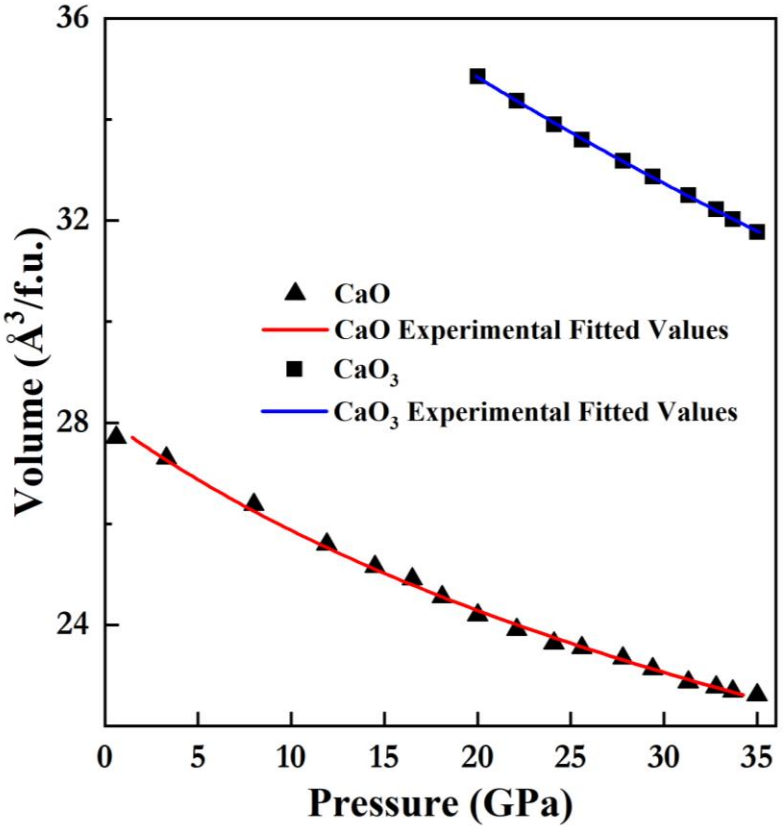

Supplementary Figure 6| Equation of state of CaO and CaO_3. Experimental volume versus pressure data (symbols) together with theoretical fits (lines) for CaO and CaO_3.

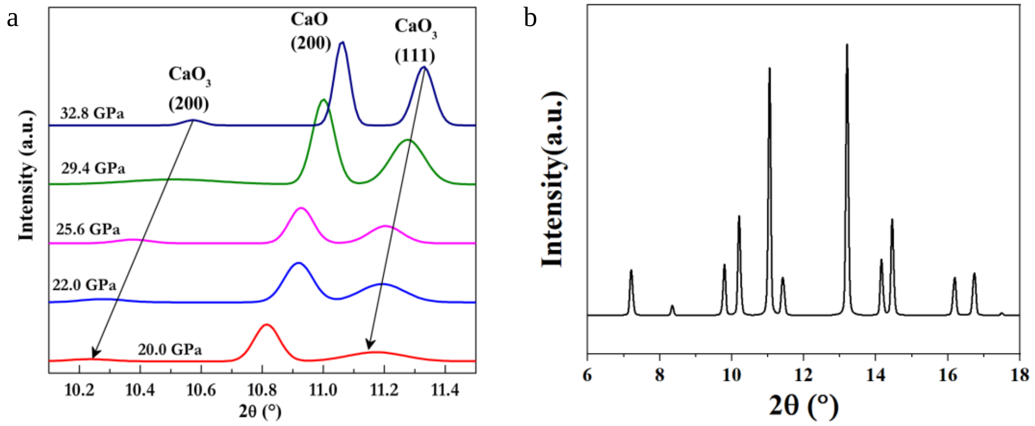

Supplementary Figure 7| XRD pattern of CaO_3 during decompression. **(a)** XRD pattern series measured during decompression of the sample. The signals for CaO_3 become weaker in intensity with decreasing pressure but clearly persists to 20.0 GPa. **(b)** The simulation of the diffraction pattern of the predicted structure of $P\text{-}42_1m$ at 35 GPa.

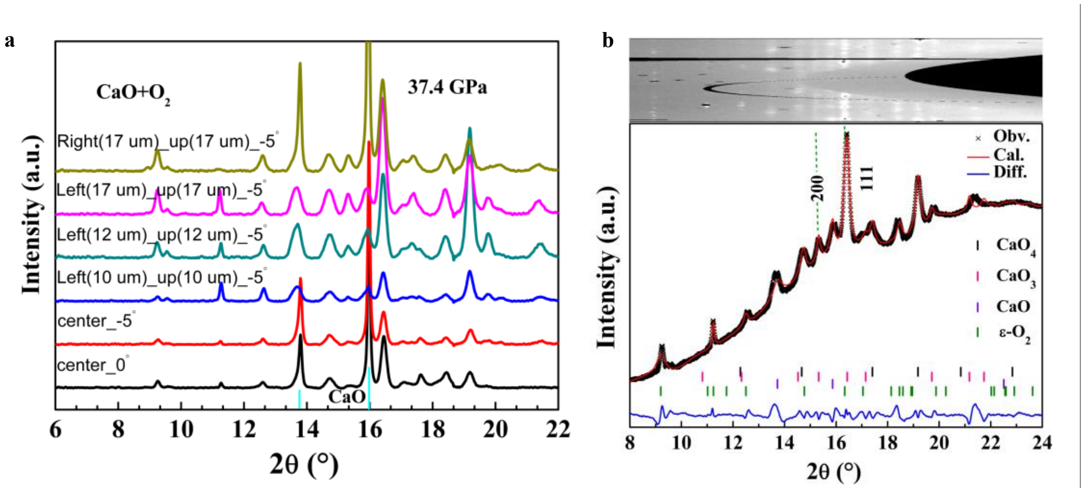

Supplementary Figure 8| Structural details from XRD. (**a)** The collected XRD patterns around heating area for Run 2 and (**b)** The Lebail fitting is performed with predicted phases at 37.4 GPa. The obtained lattice parameters are a = 4.65 Å and c = 2.89 Å for CaO_3, a = 4.10 Å and c = 4.86 Å for CaO_4.

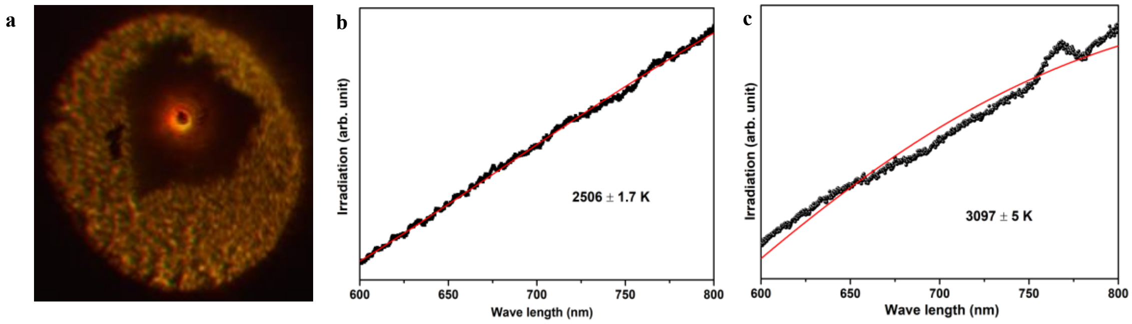

Supplementary Figure 9| Laser heating and temperature determination. (**a)** A visual image of a laser heated Fe foil. (**b)** A typical measured spectrum for Fe in DAC heated for one minute fitted by the Planck radiation function. (**c)** A manual fitting to the Planck radiation function to determine the temperature right after the CaO and oxygen reaction has occurred.

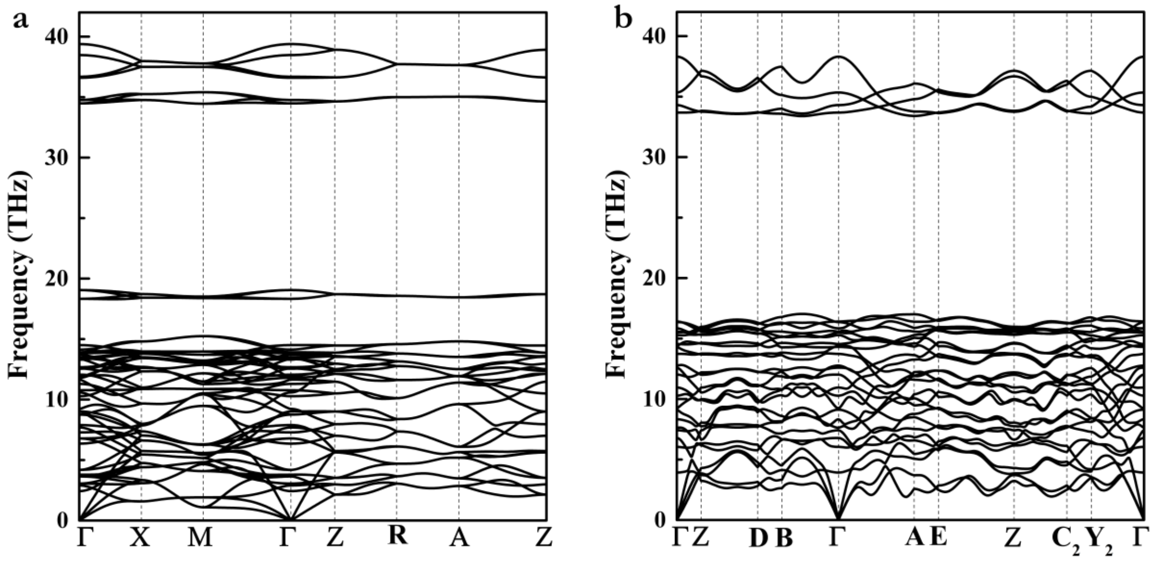

Supplementary Figure 10| Dynamic stability of CaO_4. Phonon dispersion relations along select high-symmetry points in the Brillouin zone of CaO_4 for **(a)** *I*4/*mcm* at 30 GPa and **(b)** $P2_1c$ at 50 GPa.

Supplementary References

1. Wang, Y., Lv, J., Zhu, L. & Ma, Y. Crystal structure prediction via particle-swarm optimization. *Phys. Rev. B* **82**, 094116 (2010).
2. Wang, Y., Lv, J., Zhu, L. & Ma, Y. CALYPSO: A method for crystal structure prediction. *Comput. Phys. Commun.* **183**, 2063 (2012).
3. Wang, Y. & Ma, Y. Perspective: Crystal structure prediction at high pressures. *J. Chem. Phys.* **140**, 040901 (2014).
4. Wang, Y. *et al.* Materials discovery via CALYPSO methodology. *J. Phys. Condens. Matter* **27**, 203203 (2015).
5. Lv, J., Wang, Y., Zhu, L. & Ma, Y. Predicted Novel High-Pressure Phases of Lithium. *Phys. Rev. Lett.* **106**, 015503 (2011).
6. Zhu, L. *et al.* Substitutional alloy of Bi and Te at high pressure. *Phys. Rev. Lett.* **106**, 18 (2011).
7. Wang, Y. *et al.* High pressure partially ionic phase of water ice. *Nat. Commun.* **2**, 563 (2011).
8. Wang, X. *et al.* Cagelike Diamondoid Nitrogen at High Pressures. *Phys. Rev. Lett.* **109**, 175502 (2012).
9. Zhu, L., Liu, H., Pickard, C. J., Zou, G. & Ma, Y. Reactions of xenon with iron and nickel are predicted in the Earth's inner core. *Nat. Chem.* **6**, 644 (2014).
10. Li, Y., Hao, J., Liu, H., Li, Y. & Ma, Y. The metallization and superconductivity of dense hydrogen sulfide. *J. Chem. Phys.* **140**, 174712 (2014).
11. Peng, F. *et al.* Hydrogen Clathrate Structures in Rare Earth Hydrides at High Pressures: Possible Route to Room-Temperature Superconductivity. *Phys. Rev. Lett.* **119**, 1 (2017).
12. Guillaume, C. L. *et al.* Cold melting and solid structures of dense lithium. *Nat. Phys.* **7**, 211 (2011).
13. Stavrou, E. *et al.* Synthesis of Xenon and Iron-Nickel Intermetallic Compounds at Earth's Core Thermodynamic Conditions. *Phys. Rev. Lett.* **120**, 96001 (2018).
14. Ahart, M. *et al.* Evidence for Superconductivity above 260 K in Lanthanum Superhydride at Megabar Pressures. *Phys. Rev. Lett.* **122**, 27001 (2019).
15. Kresse, G. & Furthmüller, J. Efficient iterative schemes for ab initio total-energy calculations using a plane-wave basis set. *Phys. Rev. B* **54**, 11169 (1996).
16. Perdew, J. P., Burke, K. & Ernzerhof, M. Generalized Gradient Approximation Made Simple. *Phys. Rev. Lett.* **77**, 3865 (1996).
17. Blöchl, P. E. Projector augmented-wave method. *Phys. Rev. B* **50**, 17953 (1994).
18. Monkhorst, H. J. & Pack, J. D. Special points for Brillouin-zone integrations. *Phys. Rev. B* **13**, 5188 (1976).
19. Togo, A., Oba, F. & Tanaka, I. First-principles calculations of the ferroelastic

transition between rutile-type and $CaCl_2$-type SiO_2 at high pressures. *Phys. Rev. B* **78**, 134106 (2008).

20. Heyd, J., Scuseria, G. E. & Ernzerhof, M. Hybrid functionals based on a screened Coulomb potential. *J. Chem. Phys.* **118**, 8207 (2003).
21. Bader, R. F. W. A quantum theory of molecular structure and its applications. *Chem. Rev.* **91**, 893 (1991).
22. Fonari, A. & Stauffer, S. *vasp_raman.py*. (https://github.com/raman-sc/VASP/, 2013).
23. Porezag, D. & Pederson, M. R. Infrared intensities and Raman-scattering activities within density-functional theory. *Phys. Rev. B* **54**, 7830 (1996).
24. Louail, L., Krachni, O., Bouguerra, A. & Ali Sahraoui, F. Effect of pressure on structural and elastic properties of alkaline-earth oxide CaO. *Mater. Lett.* **60**, 3153 (2006).
25. Ma, Y., Oganov, A. R. & Glass, C. W. Structure of the metallic ζ -phase of oxygen and isosymmetric nature of the ε−ζ phase transition: Ab initio simulations. *Phys. Rev. B* **76**, 064101 (2007).
26. Leinenweber, K., Partin, D. E., Schuelke, U., O'Keeffe, M. & Von Dreele, R. B. The Structure of High Pressure $Ca(OD)_2$ II from Powder Neutron Diffraction: Relationship to the ZrO_2 and EuI_2 Structures. *J. Solid State Chem.* **132**, 267 (1997).
27. Kuhs, W. F., Finney, J. L., Vettier, C. & Bliss, D. V. Structure and hydrogen ordering in ices VI, VII, and VIII by neutron powder diffraction. *J. Chem. Phys.* **81**, 3612 (1984).
28. Funamori, N. High-Pressure Transformation of Al_2O_3. *Science* **278**, 1109 (1997).
29. Suzuki, A., Ohtani, E. & Kamada, T. A new hydrous phase δ-AlOOH synthesized at 21 GPa and 1000 °C. *Phys. Chem. Miner.* **27**, 689 (2000).
30. Hu, Q. *et al.* FeO_2 and FeOOH under deep lower-mantle conditions and Earth's oxygen–hydrogen cycles. *Nature* **534**, 241 (2016).
31. Hemley, R. J. *et al.* Static compression of H_2O-ice to 128 GPa (1.28 Mbar). *Nature* **330**, 737 (1987).
32. Lu, C. & Chen, C. High-Pressure Evolution of Crystal Bonding Structures and Properties of FeOOH. *J. Phys. Chem. Lett.* **9**, 2181 (2018).
33. Nelson, J. R., Needs, R. J. & Pickard, C. J. Calcium peroxide from ambient to high pressures. *Phys. Chem. Chem. Phys.* **17**, 6889 (2015).
